# Supplementary material for: Periodontal Regenerative Therapy Using rhFGF-2 and Deproteinized Bovine Bone Mineral versus rhFGF-2 Alone: 4-Year Extended Follow-Up of a Randomized Controlled Trial
Source: Biomolecules. 2022 Nov 12;12(11):1682. doi: 10.3390/biom12111682 (PMC9688011; doi:10.3390/biom12111682)
Supplement: Supplementary file 1 [file biomolecules-12-01682-s001.zip › Supplementary tables Saito 20221102.pdf]

**Supplementary tables**

Table S1. Participant demographics and baseline parameters

| | rhFGF-2 | rhFGF-2 + DBBM | Difference |
|---|---|---|---|
| Age (years; mean ± SD) | 51.0 ± 10.2 (range, 30-69) | 52.1 ± 10.2 (range, 36-74) | N.S. |
| Gender | | | N.S.[a] |
| Men | 5 | 4 | |
| Women | 9 | 8 | |
| No. of teeth (mean ± SD) | 25.3 ± 3.8 | 25.9 ± 2.4 | N.S. |
| Clinical attachment level (CAL) (mm; mean ± SD) | | | |
| Full-mouth | 3.31 ± 0.58 | 3.30 ± 0.61 | N.S. |
| Reference site[b] | 7.07 ± 1.56 | 7.57 ± 1.64 | N.S. |
| Probing pocket depth (PPD) (mm; mean ± SD) | | | |
| Full-mouth | 2.90 ± 0.51 | 3.01 ± 0.50 | N.S. |
| Reference site[b] | 6.03 ± 1.34 | 6.31 ± 1.24 | N.S. |
| Gingival recession (GR) (mm; mean ± SD) | | | |
| Reference site[b] | 1.20 ± 1.49 | 1.24 ± 1.36 | N.S. |
| BOP positive (%) | | | |
| Reference site[b] | 72.4 | 77.1 | N.S.[a] |
| Tooth mobility (TM) (mean ± SD) | | | |
| Reference tooth[b] | 0.17 ± 0.40 | 0.21 ± 0.42 | N.S. |

Differences were assessed by the Mann-Whitney U test ([a]Fisher's exact test).
[b]n=16 per group

Table S2. Distribution of clinical attachment level (CAL) gains in the treated sites at 4 years postoperatively.

| | sites (%) | |
|---|---|---|
| CAL gain subgroup (mm) | rhFGF-2 (control, n=16) | rhFGF-2 + DBBM (test, n=16) |
| 0.0 – 1.5 | 5 (31.2) | 2 (12.5) |
| 2.0 – 4.0 | 8 (50.0) | 10 (62.5) |
| > 4.0 | 3 (18.8) | 4 (25.0) |

Table S3. Correlations between postoperative clinical attachment level (CAL) gains at 4 years and variables at baseline (post-IP).

| Baseline variable | rhFGF-2 (control) | | rFGF-2 + DBBM (test) | |
|---|---|---|---|---|
| | *r* | *p* | *r* | *p* |
| Patient age (yrs) | -0.314 | 0.237 | -0.067 | 0.807 |
| Number of teeth | ***0.602*** | ***0.014*** | -0.234 | 0.384 |
| CAL (mm) | ***0.613*** | ***0.012*** | ***0.831*** | ***0.001*** |
| PPD (mm) | ***0.611*** | ***0.012*** | ***0.664*** | ***0.005*** |
| TM | -0.166 | 0.540 | 0.123 | 0.651 |
| Defect depth (mm) | 0.369 | 0.160 | ***0.732*** | ***0.001*** |
| Defect width (mm) | 0.278 | 0.298 | 0.014 | 0.958 |

*r*, Spearman coefficient. Significant differences are indicated in bold italics.

PPD, probing pocket depth; TM, tooth mobility

Table S4. Multiple regression analysis with clinical attachment level (CAL) gains from baseline (post-IP) to 4 years as dependent variable: rhFGF-2 (control) group.

| Baseline variables | Coefficient | Confidence interval | *t* ratio | *p* |
|---|---|---|---|---|
| PPD (mm) | -0.154 | −1.158 to 0.850 | 0.342 | 0.740 |
| Number of teeth | 0.071 | −0.101 to 0.249 | 0.895 | 0.392 |
| BOP (+ or −) | 0.274 | −1.172 to 1.720 | 0.422 | 0.682 |
| Defect depth (mm) | −0.223 | −0.836 to 0.390 | 0.802 | 0.440 |

Dependent variable: CAL gain at 4 years postoperatively. $R^2 = 0.466$

PPD, probing pocket depth; BOP, bleeding on probing

Table S5. Multiple regression analysis with clinical attachment level (CAL) gains from baseline (post-IP) to 4 years as dependent variable: rhFGF-2 + DBBM (test) group.

| Baseline variables | Coefficient | Confidence interval | *t* ratio | *p* |
|---|---|---|---|---|
| PPD (mm) | 0.647 | 0.283 to 1.012 | 3.906 | ***0.003*** |
| Number of teeth | −0.008 | −0.194 to 0.178 | 0.097 | 0.925 |
| BOP (+ or −) | −0.464 | −1.437 to 0.510 | 1.048 | 0.317 |
| Defect depth (mm) | 0.703 | 0.278 to 1.128 | 3.642 | ***0.004*** |

Dependent variable: CAL gain at 4 years postoperatively. $R^2 = 0.789$

PPD, probing pocket depth; BOP, bleeding on probing
