# Supplementary figures and images for: Periodontal Regenerative Therapy Using rhFGF-2 and Deproteinized Bovine Bone Mineral versus rhFGF-2 Alone: 4-Year Extended Follow-Up of a Randomized Controlled Trial
Source: Biomolecules. 2022 Nov 12;12(11):1682. doi: 10.3390/biom12111682 (PMC9688011; doi:10.3390/biom12111682)

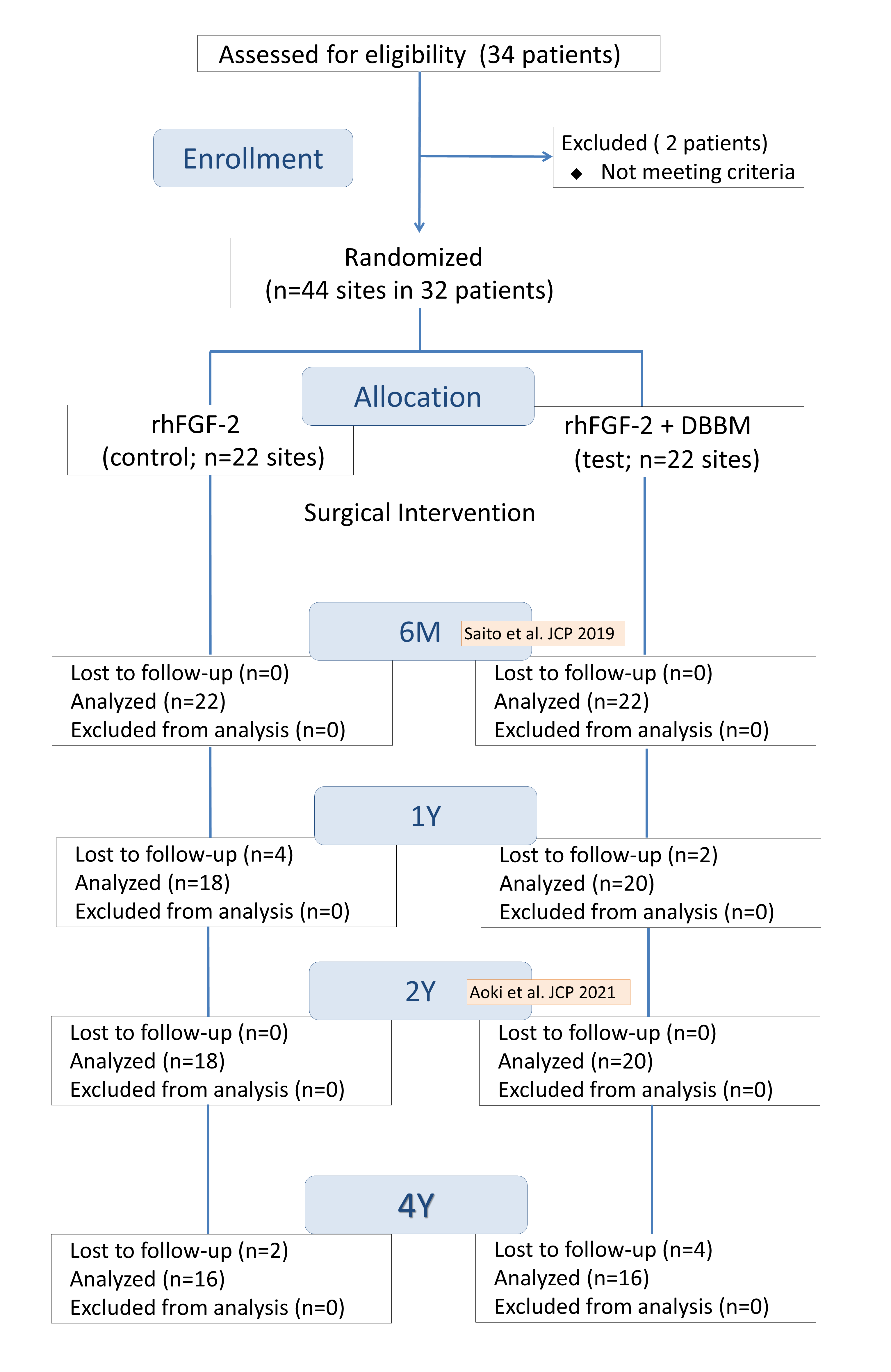

Supplement: Supplementary file 1 [file biomolecules-12-01682-s001.zip › Figure S1 Flowchart 4Y.tif]
